# Factors influencing performance of health workers in the management of seriously sick children at a Kenyan tertiary hospital - participatory action research

**DOI:** 10.1186/1472-6963-14-59

**Published:** 2014-02-07

**Authors:** Grace W Irimu, Alexandra Greene, David Gathara, Harrison Kihara, Christopher Maina, Dorothy Mbori-Ngacha, Dejan Zurovac, Santau Migiro, Mike English

**Affiliations:** 1 Department of Paediatrics and Child Health, College of Health Sciences, University of Nairobi, P.O. Box 19676-00202, Nairobi, Kenya; 2 Centre for Geographic Medicine Research – Coast, KEMRI/Wellcome Trust Research Programme, P.O. Box 230 Kilifi and P.O. Box 43640-00100, Nairobi, Kenya; 3 Child Health, University of Dundee, Dundee, UK; 4 Kenyatta National Hospital, P.O. Box 20723-00202, Nairobi, Kenya; 5 Centre for Tropical Medicine, Nuffield Department of Clinical Medicine, University of Oxford, CCVTM, Oxford OX3 7LJ, UK; 6 Center for Global Health and Development, Boston University, Boston, MA 02118, US; 7 Division of Child Health, Ministry of Health, Nairobi, Republic of Kenya; 8 Department of Paediatrics, University of Oxford, Oxford, UK

**Keywords:** Clinical audits, Clinical practice guidelines, Continuous medical educational sessions, ETAT+, Ethnographic study, Implementation of best-practices, Interpretive description, Participatory action research, Participant observer, Performance of health workers

## Abstract

**Background:**

Implementation of World Health Organization case management guidelines for serious childhood illnesses remains a challenge in hospitals in low-income countries. Facilitators of and barriers to implementation of locally adapted clinical practice guidelines (CPGs) have not been explored.

**Methods:**

This ethnographic study based on the theory of participatory action research (PAR) was conducted in Kenyatta National Hospital, Kenya’s largest teaching hospital. The primary intervention consisted of dissemination of locally adapted CPGs. The PRECEDE-PROCEED health education model was used as the conceptual framework to guide and examine further reinforcement activities to improve the uptake of the CPGs. Activities focussed on introduction of routine clinical audits and tailored educational sessions. Data were collected by a participant observer who also facilitated the PAR over an eighteen-month period. Naturalistic inquiry was utilized to obtain information from all hospital staff encountered while theoretical sampling allowed in-depth exploration of emerging issues. Data were analysed using interpretive description.

**Results:**

Relevance of the CPGs to routine work and emergence of a champion of change facilitated uptake of best-practices. Mobilization of basic resources was relatively easily undertaken while activities that required real intellectual and professional engagement of the senior staff were a challenge. Accomplishments of the PAR were largely with the passive rather than active involvement of the hospital management. Barriers to implementation of best-practices included i) mismatch between the hospital’s vision and reality, ii) poor communication, iii) lack of objective mechanisms for monitoring and evaluating quality of clinical care, iv) limited capacity for planning strategic change, v) limited management skills to introduce and manage change, vi) hierarchical relationships, and vii) inadequate adaptation of the interventions to the local context.

**Conclusions:**

Educational interventions, often regarded as ‘quick-fixes’ to improve care in low-income countries, may be necessary but are unlikely to be sufficient to deliver improved services. We propose that an understanding of organizational issues that influence the behaviour of individual health professionals should guide and inform the implementation of best-practices.

## Background

The need for improving health workers’ practice in low-income countries (LICs) has been frequently demonstrated in international and local surveys assessing the quality of care for the sick child. These studies identified poor compliance with evidence-based standards for care as some of the problems facing paediatric service delivery [1-3]. Consequently the Ministry of Health, Kenya, developed ‘Basic Paediatric Protocols’ in an attempt to introduce best-practices for emergency and early admission in-patient management of major causes of childhood illnesses. The protocols comprised of clinical practice guidelines (CPGs) adapted from World Health Organization and local disease specific guidelines [4] and originating from consultation with senior paediatricians from the University of Nairobi, Kenyatta National Hospital (KNH) and the Ministry of Health in 2005 [4,5]. From 2006 they started to become available to junior clinicians in the university and KNH with availability increasing in parallel with increased provision of ‘Emergency Triage Assessment and Treatment *Plus* admission care’ (ETAT+) training so that by 2008 most junior and senior clinicians had copies of the CPGs. The ETAT+ training itself is a 5-day programme for dissemination of the CPGs developed to support their implementation [4,5]. Course design draw on educational theory and, for content, on the WHO’s Emergency Triage Assessment and Treatment (ETAT) course as well as the evidence-based CPGs [5,6].

The CPGs and ETAT+ focus on the emergency and early admission care for children and target all cadres of health workers although much of the focus is on clinical assessment, diagnosis and management [4]. Though primarily aimed at district hospitals, demand for them grew in KNH, a tertiary care facility and teaching hospital for University of Nairobi Medical School. Initially this resulted in *ad hoc* delivery of ETAT+ training from 2006. Later this was formalized by the hospital and the university with a steady increase in training coverage so that by July 2008 over 90% of the trainee paediatricians, two thirds of the clinical officers and the consultants, and a third of the nurses who provided care for the seriously sick child in KNH had received the CPGs and ETAT+ training [7].

However, prior research suggests that dissemination of printed materials and training do not produce large changes in actual practice [8,9]. We were interested to explore what additional strategies would be acceptable to KNH staff to improve the uptake of best-practice recommendations. Drawing on the PRECEDE-PROCEEED model [10] as a guiding framework we postulated that audit and feedback and continuing medical education sessions (CMEs) might be effective reinforcement strategies. To facilitate introduction of these reinforcement strategies, to understand their use and to explore their value we adopted a participatory action research (PAR) approach [11,12].

The PAR applied is complemented by linked quantitative reporting that evaluated the impact of these implementation efforts on adoption of recommended health care practices in KNH against quality indicators agreed upon by the staff [7]. In brief, at baseline (in 2005) patients’ care was largely inconsistent with the national and international clinical guidelines, with nine out of 15 key indicators having performance below 10%. The dissemination of the CPGs and ETAT+ accompanied by efforts to introduce audit and feedback and CMEs resulted in considerable improvements in adherence to a number of the guidelines recommendations. We observed an absolute effect size of over 20% improvement in seven out the 15 key indicators. However, the improvements varied across diseases and with time, and for five of the indicators performance was below 10% in the post-intervention period (2009). In this paper, we describe how strategies to promote uptake of the CPGs and ETAT+ evolved with a focus on audit and feedback and CMEs. We explore the facilitators of and barriers to implementation of best-practices with the aim of helping guide future implementation efforts in similar settings.

## Methods

### Study site

KNH is a State Corporation whose vision is ‘To be a regional center of excellence in the provision of innovative and specialized health care’. It is a national referral hospital and the teaching hospital for the School of Medicine of University of Nairobi (UoN) and other medical training institutions. KNH has a bed capacity of 1,800. There are 14,000 paediatric admissions annually to four general paediatric wards each with 60 beds. The bed occupancy is often over 100% and all patients are charged user fees. Our work focused on clinical care of children aged 2 to 59 months admitted to hospital with pneumonia, diarrhoea and severe malnutrition. Our specific interest was in the care delivered in the first 48 hours, the focus period of the CPGs and ETAT+ [4,7].

Most of clinical in-patient care is provided by 60-75 trainee paediatricians enrolled in a three-year postgraduate paediatric training programme through the UoN. They are normally supervised by 25 paediatricians, out of whom 15 are academics from the university. The paediatricians are highly qualified with 22/25 being professors or having paediatric subspecialty training (e.g. in cardiology or nephrology) in line with the hospital’s vision. The KNH clinicians are answerable to the KNH head of paediatric clinical services, while the academics and the trainee paediatricians are answerable to the Chairman of the Department of Paediatrics, UoN. There are 126 qualified nurses on the general paediatric wards; twelve to twenty nurses per working shift to cover the 240 bed paediatric unit.

### Study design

This was a hospital-based pragmatic, ethnographic study based on the theory of participatory action research.

### Data collection and participants

We utilized the participant observation approach for data collection. We chose this approach because we aimed to understand group culture and have direct experiential and observational access to the participants’ world of meaning [13]. This approach allows access to subliminal and subconscious forms of knowledge expressed as behaviour that resist and defy linguistic translation. We utilized naturalistic inquiry to obtain information from all hospital staff encountered; therefore avoided introducing bias by selecting only the staff willing to participate. In addition, theoretical sampling was applied to allow in-depth exploration of emerging issues through more focused observation and informal discussions [14]. We elected not to use formal, scheduled interviews preferring the continuous exploration possible with the 18 months of participant observation.

Data were collected by one of the researchers (GI) who also played the role of a participant observer (PO) and facilitated the PAR. She kept a field diary over 18 months as a repository for her observations, memos and reflections and took still photographs of relevant scenes such as treatment sheets and patients’ notes. She held and made notes of opportunistic conversations with the staff and obtained information from secondary data such as hospital and Ministry of Health policies. In this study, audio-taping or diary recording in real-time was not applied as this was felt to inhibit the staff expressing themselves. Thus, the PO made rapid field notes that were expanded into proper diary entries every evening. Consequently, we have no verbatim quotes; rather we have (and present as illustrative data) excerpts from the field diary representing recollection of observations and conversations.

### Panel 1: Background of the participant observer (PO)

One of the authors (GI) took a participant observer role. Her role was determined by the local context, her background knowledge and experiences. In brief, we saw her as a permanent insider of KNH. She was a paediatrician who trained in KNH, after which she was employed as an academic in the UoN’s School of Medicine and honorary paediatric consultant in KNH. Her status enabled her to interact closely with most of the front-line service providers in all the paediatric units. Being an insider, the PO could use internal jargon and draw on her experience while speaking to her colleagues, as well as following up on their responses to enrich the data.

She participated in the development of the CPGs and ETAT+ course and subsequently in a cluster randomized trial that evaluated their impact in district hospitals [4,15]. She had previously conducted qualitative research [16-20]. Her background enabled her to take participatory roles in different capacities; as a consultant paediatrician, academic, ETAT+ trainer and as a researcher conducting an action oriented ethnographic study.

### Our assumptions at the time of designing this research

We assumed that KNH had established structures to allow adoption of best-practice recommendations. For example, structures to allow CMEs and clinical audit activities. We anticipated that these activities would be supported by the paediatricians particularly those trained in ETAT+. Based on these assumptions we used the concepts of the PRECEDE-PROCEED health education model as the conceptual framework to guide and examine further reinforcement activities to improve the uptake of best-practices in KNH. The acronym PRECEDE stands for Predisposing, Reinforcing and Enabling Constructs in Education/Environment Diagnosis and Evaluation. This model was chosen because it allowed designing reinforcement strategies that were strategically planned to meet demonstrated needs (Table 1). It also recognized the importance of the participants in defining their own high-priority problems and goals in developing and implementing solutions [10].

**Table 1:** Definition of predisposing, enabling and reinforcing factors and the strategies employed to influence them

	**Definition (Adapted from Green et al)‡**	**Strategies employed to influence the factors**
*Predisposing factors*	Factors that improve care providers’ knowledge, existing skills, values, attitudes, beliefs, personal preferences and self-efficacy towards desired change in practice.	Creating awareness of the gap between current practices and expected practices, enhancing staff’s knowledge and skills, and promoting ownership of the quality initiatives.
*Enabling factors*	Psychological, emotional or physical factors in the local context that would facilitate motivation to change behaviour.	i) Skill enhancement e.g. using CPGs to aid in clinical decision-making, ii) engaging staff in identifying problems and feasible solutions at all levels, iii) provision of basic resources, iv) better organization of service delivery and, v) encouraging the front-line service providers to do things differently to improve service efficiency.
*Reinforcing factors*	Factors that strengthen the motivation to perform the desired action [10].	Making the staff aware of the progress of implementation of the quality initiatives, making their progress visible, having them identify with the initiatives by involving them in problem-solving and action planning sessions.

‡Green L, Kreuter M, Deeds S, Partridge (Eds.): Health education planning: A diagnostic approach: Mayfield Press; 1980.

### Data analysis

Diary data were analysed using interpretive description. Interpretive description was preferred to other approaches because it recognizes that reality is complex, contextual and constructed. It also allows the prior knowledge the researchers have based on experience, education, training and personality to be drawn on [21].

The process of data analysis was ongoing during data collection for the purpose of theoretical sampling and saturation [14]. After completion of the PAR, data were reanalysed to develop a deeper, more holistic understanding and interpretation of the findings. This was facilitated by repeatedly reading all the data to achieve immersion. With study objectives and emerging issues in mind the data were re-read word by word, highlighting chunks of text that addressed the key questions. The data were then coded manually focusing on incident coding. The definition of these codes evolved inductively. These codes were verified by two of the researchers (ME, AG) who were not involved in the initial coding. A reflective approach was used to allow constant exploration of related questions such as: ‘What is happening here? Why is this happening? Why not something else? What does it mean to the health worker, organization and to the patient? Is there a dialectic relationship between what the data are telling us and information in the KNH policies and other secondary data?’

Concepts were developed that were compared with more empirical frameworks and with each other to sharpen their definitions and define their properties. Similar concepts were grouped together to form categories and subcategories identified. Linkages were then made among the various categories by identifying the core themes around which all the other categories were subsumed. In this analysis, we draw on social cognitive theory [22,23] and theory on complex adaptive systems [24] to explore broadly factors that influenced uptake of best-practices in this complex environment.

### Enhancing reflexivity

During audit feedback, problem-solving meetings and CMEs, the PO explicitly and deliberately allowed the participants to consciously reflect on emerging interpretive insights to enhance reflexivity. Further, a preliminary analysis and interpretation was the subject of discussions with a group of social scientists who were not directly involved in this research and subsequently with key people in KNH and UoN. These discussions helped in data verification, testing face validity and ensured that our analysis was grounded in a broader understanding of how systems change.

### Ethics statement

Ethical approval was provided by the Kenyatta National Hospital/University of Nairobi Ethics and Research Committee (reference number KNH-ERC/01/480). This study was classified as a field/observational study and informed consent from the participants was not found necessary by the institutional ethics review committee because research could not be effectively carried out if consent were obtained. The participants’ confidentiality has been preserved.

## Results

We first present the evolution of the reinforcement activities during the PAR before presenting our understanding of the barriers to and facilitators of the implementation process that shaped this evolution. Excerpts from the field diary are embedded within the results section, as illustrations of our findings.

### Evolution of the participatory action research

In the early stage of PAR we build capacity for staff participation and developed quality indicators against which health workers’ performance could be evaluated. We describe the evolution of reinforcement activities within eighteen months (June 2008-December 2009) of PAR focussed on institutionalization of clinical audits and addressing gaps in knowledge and skills.

### Panel 2: Promoting staff participation

An initial aim was to engage KNH staff in the action oriented process from the stage of proposal writing and promote their participation. We formed a core-group that comprised of 12 key decision makers; six nurse managers, four senior clinicians (two in authoritative positions), one medical records personnel and one of the researchers (GI). These were purposively selected for their potential to coordinate quality initiatives. We enabled the KNH staff to participate in this research by enhancing their knowledge and skills through ETAT+ training. The training was largely planned and financed by the hospital management. Our next goal was to develop a locally acceptable approach to routine assessment of care against quality indicators (QIs) considered feasible in KNH. Lastly, using a participatory approach, we aimed to identify problems in service delivery and, feasible and acceptable action plans to improve care in a collaborative manner.

*Development of quality indicators:* Candidate QIs were adopted from ETAT+ and CPGs and targeted three diseases: - pneumonia, diarrhoea and severe malnutrition. Adaptation of candidate QIs to the KNH context was initially done by 12 panellists who included four nurses, two clinical officers and six doctors (four from UoN) who were all ETAT+ trained and had shown prior interest in quality initiatives. Initially each person was given a questionnaire and asked to indicate if the candidate QIs were: i) applicable to all the targeted patients, ii) feasible to assess from case records and, iii) linked to better outcomes defined by improved chances of correct diagnostic classification, survival or shorter hospital stay. They were encouraged to consult their colleagues. Only three panellists completed the questionnaire within the allocated time of one month. Others said they had misplaced it or they were very busy while still expressing interest in the exercise. Subsequently, a face to face meeting that utilized a consensus method adapted from the nominal group technique [18] was held to identify QIs. The meeting was attended by eight of the original 12 panellists and four new members. The meeting was moderated by a senior paediatrician (DM), an experienced moderator, who gave people an opportunity to express themselves regardless of their professional background. One of the researchers (GI) was present both to provide information on the scientific evidence behind the QIs when it was required and as an observer of the process. The QIs agreed upon by KNH staff spanned four domains of care: assessment (n = 24), classification (n = 3), treatment (n = 6) and monitoring of patients in the first 48 hours of admission (n = 7).

This initial process to develop and then disseminate the QIs provided the first suggestions that staff were often unfamiliar with the link between evidence and quality indicators and, more generally, lacked awareness that quality of care (QoC) might be poor.

### Institutionalizing clinical audits

We report four chronological phases of attempts to use clinical audit as a tool to identify problems and develop feasible solutions and action plans. These indicate the difficulty one may have in implementing audit and applying the Plan-Do-Study-Act cycle as a reinforcement strategy.

i) *Re-energising routine audit* (*June to August 2008*): While hospital policy was that clinical audits should be done every two weeks, they were however done irregularly and focused mainly on simple descriptions of mortality rates. The core-group recommended use of more regular audits with a focus on improvement using the ETAT+ audit tool for ‘problem based mortality audit’ [5]. The KNH Quality Assurance Unit was tasked to coordinate the audits. Unfortunately there was no follow-up or reinforcement of this core group recommendation and routine audits remained unchanged.

ii) *Facilitation of ward-level clinical audits* (*September to October 2008*): After three months of limited activity it was agreed that the PO should facilitate clinical audits at the ward level. The trainee paediatricians were supportive in preparation of audit reports. However, they were neither experienced nor skilled in giving feedback to ward teams and inadequate engagement of the academics was a persistent challenge. There were also concerns that the audit tool was very detailed, and thus time-consuming. Ward-based clinical audits therefore failed to become routine unless the PO consistently organised and facilitated these meetings making this approach unsustainable, as the following statement suggests:

‘No, I have no time. You concentrate on some of these things and have no time to do university duties. Besides there are no resources to provide quality care’. (Senior *academic; -response to invitation to attend a ward mortality meeting*).

Despite being unsustainable, ward-level audits did reveal suboptimal patient care such as inadequate patient assessment, misdiagnosis, incorrect treatment prescriptions, and failure to administer treatments or review and monitor patients’ progress. During feedback meetings it was also apparent that there were gaps in problem identification and problem-solving skills. For example, preoccupation with workload and patient congestion on the wards, only to be solved by an increase in staff numbers precluded discussion of efforts to improve staff competence or service organization to improve efficiency.

iii) *Departmental level audits initiated* (*November 2008 to June 2009*): The core group acknowledged failure of ward-level audits and proposed that audit be coordinated at a central point. In this third approach, we aimed to develop a simple audit tool with dichotomous responses reflecting quality indicators (QIs) achieved (or not) to be used by an audit team. It was proposed that this team should consist of six nurse managers, a hospital administrator, a representative of the trainee paediatricians and four paediatricians (including two academics). The academics were not formally informed and minutes of the meeting were not kept therefore undermining active follow-up. A simple audit tool and its corresponding standard operating procedures were nevertheless successfully developed and an audit, described by the staff as an ‘eye-opener’, was conducted in March 2009.

An audit feedback meeting was held that was attended by KNH staff including nine consultants though only two were from UoN. The audit revealed a limited awareness of critical patient safety issues, such as wrong or missed diagnoses, drug errors and lack of evidence that treatment prescribed was given. The audit-feedback was supported by photographs of relevant evidence. Audit criteria were explained and process maps were used to make issues more real to the staff. The feedback was followed by a problem-solving and action planning session.

Although the audit feedback was in many ways successful at raising awareness of shortcomings in care, the issue of staff shortages, notably nursing staff shortages, dominated the discussion. Some of the consultants argued that nurses were too overworked to provide better care (nothing can be done) while others argued for prioritization of care for seriously sick patients, as reflected here:

*‘*Yes, we all agree there is acute shortage of nurses, but the issue we are discussing is prioritization of care. Even at home, for example, if there is not enough food, you can’t say everyone will not eat, you plan what you can afford, but ensure that the young children get enough if possible. It is the same way we should prioritize care of the very sick patients’ (*KNH paediatrician*).

While conducting this audit it also became clear that only the trainee paediatricians and the paediatricians were sufficiently knowledgeable to collect the necessary clinical data, though they required constant reminders to use the SOPs to ensure consistent evaluation.

Although it finally appeared that a possible mechanism had been identified for engaging staff widely in audit, no other departmental-level medical audits were conducted. There appeared no broader leadership to champion the process with the continued expectation of the hospital management that the PO would be responsible for this area. Perhaps because the PO had worked in KNH for a long time most staff, and the management too, regarded her as an insider rather than a facilitator/researcher. However, devolution of the process came with neither substantial support nor authority. Further, although during audit feedback there was a problem-solving session with an action-plan developed, hardly any action was subsequently implemented and there was insufficient monitoring of proposed action. As staff attributed most of the problems to a shortage of resources and facilities, the next approach tried was to combine audit of the process of care with assessment of the structure in which care was delivered.

iv) *Abandoning clinical audit for a broader hospital survey approach*: In this fourth approach we adapted a survey tool developed for rapid hospital assessment that combined audit of the process of care with assessment of the structure in which care was delivered – helping to address staff concerns over resource availability [5]. A two-day rapid hospital assessment conducted by a panellist of six members nominated from the departmental level audit team (two nurse managers, hospital administrator, a personnel from the KNH Quality Assurance Department and two senior KNH paediatricians) and facilitated by the PO was undertaken in July 2009. Only two of the panellists participated in the entire survey, rest of the team joined the survey briefly at their convenience, undermining our intention to build staff capacity.

During the feed-back meeting, the staff identified problems in the entire continuum of care from assessment to monitoring of patients. In fact, the audit team was concerned about staff reaction and feared that the feedback could be de-motivating, as this example shows:

*‘*Feedback should be presented in a manner that staff don’t feel they are being policed, rather just making them feel even if there is a gap in care, they can manage to improve. Otherwise they can rebel and give-up.’ (*Member of audit team*).

Inadequate knowledge and shortage of resources compounded by inability to prioritize care for the seriously sick patients were cited as major problems. This audit approach was deemed to be feasible and it was agreed that time was required for implementation of proposed actions before re-evaluating service delivery. A follow-up hospital survey was planned for January 2010, after 6 months, but two and half years later no further survey had been carried out.

In summary, we tried four approaches to introduce routine clinical audits but none seemed sustainable. In addition to other barriers (presented below) it seems that poorly defined staff roles, insufficient commitment by management to improve quality of care (QoC) compounded by inadequate managerial skills and lack of a problem identification/problem solving culture, all contributed to this failure. This was despite linked efforts that were being made to address identified gaps in knowledge and skills, now described, to support improved technical competence.

### Addressing gaps in knowledge and skills

As attempts were being made to introduce regular audits, we addressed gaps in knowledge and skills in clinical practice in two stages. First, by providing the 5-day ETAT+ training and subsequently addressing gaps in competence identified from the audit and feedback meetings.

#### Increasing ETAT+ coverage

At the commencement of the PAR, there were regular ETAT+ trainings for medical students of UoN. It took however over one year without success to arrange any training for KNH staff. Efforts to organize the training were energized by the PAR and the hospital supported training of seventy staff from the paediatric department. This would not have been achieved, however, without the emergence of a ‘champion of change’ who negotiated with KNH management to sponsor the training, for example:

‘You see people trust me because of the changes we have made in PEU (Paediatric Emergency Unit)…; they recognize there is a gap in care… There was no problem; I just informed the training centre that the staff needed to be trained… There is money reserved for training and is usually not utilized’. (*Champion of change*).

Interestingly the meaning, value and outcome expectations linked to ETAT+ training differed between management and staff groupings. For example, trainee paediatricians had a positive outcome expectation because ETAT+ was an examinable subject. Among the nurses ETAT+ conferred prestige as the same course was taught to doctors. However, provision of care by nurses (the execution of management plans) consistent with the best-practice recommendations was not a major component of the course nor explicitly part of their performance appraisal system. Moreover, there were varied institutional goals for supporting ETAT+ training. The KNH management aimed to comply with the hospital’s directive to improve health workers’ performance through continuous professional development. UoN’s goal in incorporating ETAT+ into the curriculum was to improve the quality of the undergraduate and postgraduate paediatric programmes by teaching evidence-based paediatrics. Thus, although both institutions in theory wished to improve quality of care, it appeared that providing ETAT+ training was in itself an acceptable end point rather than a mechanism to achieve this final goal.

#### Addressing persisting gaps in knowledge and skill

The ETAT+ trainings, problem-solving sessions and changed practices of early adopters were key steps in creating awareness of the gap between what staff ought to know and what they actually knew. We initially therefore expected CMEs would focus on the ETAT+ content, with clear objectives related to achieving the quality indicators. Contrary to our expectations, assumed goals and needs for CMEs were actually different from those expressed by the recipient staff (Table 2). These unanticipated needs (such as teaching on rational use of antibiotics or management of acute asthma) took up time and effort and while valuable did not necessarily directly support implementation of the specific guidelines assessed by the quality indicators. The progress of attempts to utilise CMEs to improve patient care are now briefly summarised.

**Table 2:** Summary of the CMEs held during the study period

**Quarter, Year**	**Participants**	**Topic (number of CMEs)**
Q3, 2008	Combined ward staff^a^	Supportive care^b^ (n = 4)
Q4, 2008	PEU staff	Use of pulse oximeter (n = 1)
Q1, 2009	ETAT+ trainers	Use of pulse oximeter and skills of teaching the procedure (n = 1)
	Ward nurses	Supportive care (n =11)
	Clinicians^c^	Management of acute asthmatic attack (n = 1), Acid–base disorders (n = 1), Rational use of antibiotics (n = 1)
Q2, 2009	Ward & PEU nurses	Fluid therapy (n = 1)
	Clinicians^c^	Fluid therapy (n = 1)
	Ward nurses & nutritionist	Severe malnutrition (n = 1)
Q3, 2009	Ward nurses	Fluid therapy (n = 1), pneumonia (n = 1)
	PEU staff	Severe malnutrition (n = 2), pneumonia (n = 2), fluid therapy (n = 1)
	Clinicians^c^	Severe malnutrition (n = 3) pneumonia (n = 1)
Q4, 2009	Ward nurses & nutritionists	Severe malnutrition (n = 1)
	Biomedical staff	Oxygen therapy (n = 1)
	PEU staff	Management of acute asthmatic attack (n = 1)

^a^All the front-line service providers (nurses, clinicians and nutritionist).

^b^Oxygen therapy, intravenous fluid therapy, prevention of hypoglycaemia, interpretation of patient’s vital signs.

^c^Clinicians – trainee paediatricians and the clinical officers.

#### *Delivering CMEs*

We initially attempted ward specific CMEs and all the front-line service cadres were invited. Differences in knowledge and needs meant this approach changed to provision of cadre-specific CMEs to address procedural and basic knowledge. One consequence however was the lost opportunity for promoting cross-cadre understanding. We conducted 32 educational sessions during the 18 months period, their duration ranged from 0.5 hrs -2 hours (n = 29) to 0.5 day-1 day (n = 3) (Table 2). The fact that the staff themselves identified needs allowed delivery of CMEs that focused on very basic issues without appearing patronising to professionals. The clinicians preferred case scenario or mixed didactic and interactive formats with an emphasis on content knowledge and ‘understanding why’. Interestingly such staff felt their basic knowledge was generally adequate, despite the audit and feedback showing otherwise. The nurses liked didactic sessions followed by practical sessions to impart procedural knowledge and reflective exercises such as clinical auditing in groups to examine practices. Generally there was little interest in evidence of impact of the best-practices on the patients’ outcomes.

#### *CMEs facilitation*

The PO or the trainee paediatricians under the mentorship of the PO facilitated the nurses’ sessions. Though they were interested in the CMEs, the nurses took minimal efforts to organize their own CMEs and did not appear to make substantial effort to translate this new knowledge into action. The clinicians organized their own CMEs with minimal support by the PO. The clinicians preferred topic experts, from within and outside UoN to facilitate, though the trainee paediatricians also facilitated the sessions.

We have described how the PAR evolved. The challenges encountered are summarized in Table 3. It is noteworthy that accomplishments were largely with passive rather than active involvement of the hospital management. For example, mobilization of resources to support meetings and purchase inexpensive, essential equipment was relatively easily undertaken. However, activities that required real intellectual and professional engagement of senior staff, and their time, were a challenge. Adopting PAR as an approach became a process of sense-making and learning rather than following the pre-identified PRECEDE-PROCEED framework. The participatory nature of the study allowed the staff to decide the ‘*what and how’* of reinforcement activities. Their actions (and inaction) shaped efforts at implementation underscoring the complexity of relationships.

**Table 3:** Aims, processes and challenges of the participatory action research

**Aim**	**Process**	**Challenges**
Engagement of KNH staff	Formation of core group and involving them in implementing the best-practices.	Capacity building missed out organizational issues such as teambuilding, supervision skills, communication skills and negotiation skills.
Development of quality indicators (QIs)	Adoption of ETAT+ based QIs with targets using face to face meetings and consensus conference.	Less success for approaches requiring self-administered questioners with preference of face to face thus increasing cost of the activity.
		No preliminary study to inform performance target. Targets set at 100% correct performance based on the perceived simplicity of the tasks.
Institutionalization of audits and feedback	Re-energizing routine ward audits Facilitation of the ward audits Formation of department audit team, development of an audit tool and conducting audit. Adopting a rapid hospital survey approach to assess both structure and processes of care	Managers had insufficient skills and motivation to introduce change in a system. Minimal consultants’ support. Staff not compelled to know their clinical performance.
		Problem-solving challenged by poor culture for self-directed reading on quality care and by deeply engrained practices that had become the norm, thus difficult in recognizing suboptimal care and to do root cause analysis
		Multidisciplinary feedback that would encourage system-wide problem and solution identification was compromised by limited repertoire of knowledge on basic patients’ care that required discipline specific audit feedback details
		Insufficient structures to support the clinical audits without involvement of the facilitator
Address knowledge gaps.	Initially we held multidisciplinary educational sessions but finally adopted task oriented CMEs analogous to the format for cadre specific pre-service training.	Punctuality problems among all cadres that reflected the norm of the hospital staff. No effective learning culture, no substantive mechanism of holding the management and staff accountable for QoC
		Multi-professional capacity building not achieved due to poor communication and limited of repertoire of basic and procedural knowledge.
		No substantial incentives to attend or facilitate CMEs e.g. accreditation of CMEs

In the sections that follow we draw on our emerging understanding, derived from the PAR, of the facilitators and barriers to delivering a broadly effective intervention measured by evidence of adoption of the CPGs recommended best-practices. We illustrate the major themes identified with diary excerpts where appropriate.

### Facilitators that supported implementation of best-practices

Our analysis identified three main themes of importance that enhanced the implementation of best-practices. These were an ability to mobilize resources, the relevance of ETAT+ training to routine work and emergence of a champion of change.

#### Resource mobilization

Improving care was a shared goal of the CPG/ETAT+ approach and KNH where ETAT+ training fitted well with the planned and budgeted hospital activities. Therefore, the hospital management provided financial support to help implement best-practices. For example, the management provided resources for a consensus conference to develop the QIs, sponsored ETAT+ training and CMEs, adapted and introduced a structured paediatric admission records and supported infrastructure improvements to the oxygen delivery system. Equipment such as height measuring boards, appropriate bag-valve-mask devices and a refrigerator for storage of the milk for malnourished children were made available as soon they were found necessary. To improve care for the seriously sick patients, KNH management, in collaboration with UoN, introduced a clinical rotation for trainee paediatricians in the paediatric emergency unit to ensure coverage of the unit by a qualified doctor.

#### ETAT+ training was relevant to routine work

ETAT+ was easily integrated in the medical school curriculum and accepted as a way of updating the existing curriculum to be evidence-based. The ETAT+ instructors were mainly trainee paediatricians; they supported learning for undergraduate students and other service providers [25]. The training focused on basic aspects of routine care and did not require significant extra resources. The brevity and pocket size of the CPGs made them user-friendly among the clinicians. The structured paediatric admission records used in ETAT+ training provided a template that was adapted for use in KNH allowing the institution to rapidly gain a further success introducing its own structured paediatric admission records based on ETAT+ principles. Finally, positive outcomes observed within a short time of introduction of CPGs/ETAT+ promoted use of the CPGs as depicted in the excerpts below:

*‘*The thing (ETAT+) is working. We rarely get children dying from diarrhoea. If it happens, we ask ‘why’. (..Has the case fatality really come down?)… Oh yes, I can show you our records… you know children really used to die, especially those who were in shock… anyway we didn’t even know they were in shock’. (*Ward nurse manager*).

‘We don’t have deaths in PEU (Paediatric Emergency Unit) anymore, except those brought in dead. We manage patients well, fix IO (intraosseous) and we resuscitate… you should be there when we are resuscitating. But we get disappointed sometimes because the care on the wards is not good, some of those patients die, sometimes they don’t even get the (IV) fluids. (PEU nurse).

#### Emergence of a champion of change

A senior KNH paediatrician, who we refer to as Dr W. played a significant role soliciting support and leading quality initiatives within the paediatric department from the time the study project began; building capacity to support implementation of ETAT+ recommendations (Table 4). Dr W. had participated as an external evaluator in the project to implement the MoH CPGs in the district hospital [15]. Dr W., a senior sub-specialist, had a keen interest in common serious childhood illnesses and his role in clinical leadership was recognized within the hospital. He performed and promoted clinical procedures that were not routinely done by other paediatricians for instance establishing intra-osseous (IO) access. The following excerpt illustrates staff notions of leadership:

**Table 4:** Attributes and behaviour of the champion of change that facilitated uptake of ETAT+ recommendations in KNH

**Thematic qualities**	**Attributes and behaviour of the local champion that facilitated implementation of ETAT+ recommendations**
Led from the front	Regular supervision of staff, was visible and appreciated good performance Created learning opportunities Role model of a good clinician, actively involved in patients’ care
Overcame organizational inertia	Addressed the needs of staff (he was trusted by people because of his previous achievements in improving care and he understood the system) Took it as his personal responsibility to improve care Took risks of introducing changes which were not owned by the management and staff initially Had patience for staff as they went through stages of change Empowered others in leadership roles Believed in ability to improve care with available resources

*‘*Changes need a driver like Dr W…….you need to translate what you have learned into practice’. (*ETAT+ training closing ceremony; - Senior Administrator, KNH*).

*‘..*Encourage the staff to do the right thing any time you are there, you don’t have to be there all the time but be visible; they should feel your presence. They will do the correct thing. People are happy when they are supervised and appreciated’. *(Dr W.).*

‘You know Dr W. is always here, he has taught even the nurses to do IO *(intraosseous)*, when he is around he also does it. We call him anytime we have a difficult IV *(intravenous)* line’. *(PEU Nurse).*

In addition, Dr W. facilitated reorganization of the lay-out of the ward he was assigned to. All the severely ill children were cohorted in one room and oxygen outlets were increased from two to ten. This change was welcome by staff (see below):

*‘*I feel I have been given opportunity to think and use my knowledge to improve care. You can see even the nurses are happy with their work’. (*Nurse manager responding to question on how she feels about the new ward lay-out*).

However, despite apparent acceptance of the new ward-lay out, two of the wards lacked a champion to facilitate this change.

### Barriers to implementation of best-practices

Our analysis identified seven major themes for factors that hindered the implementation of the best-practices. These included: i) mismatch between the hospital’s vision and reality, ii) poor communication, iii) lack of objective mechanisms for monitoring and evaluating quality of clinical care, iv) limited capacity for planning strategic change, v) limited management skills to introduce and manage change, vi) hierarchical relationships, and vii) inadequate adaptation of ETAT+ to the local context.

#### Mismatch between hospital’s vision and reality

KNH strategic planning was based on its vision to provide innovative and specialized health care related to its status as a national referral hospital. However, this was in contrast with the reality that the majority of the paediatric patients were admitted with common acute illnesses that did not require ‘innovative and specialised’ health care. The situation was aggravated by weakness in the lower level health facilities that was not adequately addressed by KNH outreach services as alluded to by a senior manager ‘*when we turn patients away, they come back in worse condition and come to die here in KNH’*. Thus, KNH actually served as a national referral facility, a provincial and district hospital and, as a primary health care centre for walk-in care. This conflict of identities led to mismatch of infrastructure and skill mix of the work force did not sufficiently match needs.

#### Mismatch of infrastructure

In line with KNH’s vision to be a world class referral hospital in the provision of innovative and specialized health care, resources available were often not the most appropriate for an actual role caring for the large numbers of acutely sick children with common illnesses. Thus there was, for example, an inadequate holding area for seriously ill children where skills and resources could be concentrated. They were dispersed on the general wards among stable patients with acute or chronic illnesses.

#### Skill mix of the work force

In keeping with the hospitals’ vision, majority (22/25) of the paediatricians providing services in KNH were subspecialists or professors. We observed that many of these sub-specialists perhaps felt less obligated to focus on the management of common illnesses that they regarded largely as the concern of the trainee paediatricians or other junior staff, as illustrated by the following:

‘..during your presentation some people (referring to a senior academic) were wondering whether you were presenting to the right forum. I guess she thought it was cheap stuff’*. (An academic commenting on a presentation by the PO to academics and trainee paediatricians on ‘rational fluid therapy for dehydrated patients’*).

#### Poor communication

Poor communication took several forms and appeared compounded by a centralized administrative system and insufficient forums where working relationships could be discussed. In KNH context, centralized administrative systems were often cadre-specific. For example, paediatricians did not have substantial authority over nursing staff, as illustrated by the following excerpt:

‘…No, that (*paediatricians checking treatment charts during ward round to ensure treatment is given as prescribed*) will not work; we shall be at loggerheads with the nurses’. (*Senior academic and ward-incharge; - Trainee paediatricians’ seminar*).

Exercising authority was also undermined by lack of explicit role clarity. However, the latter was not considered a major problem. Instead, professionals were expected to be self-organizing and regulating (implied by people being referred to as adults) with the hospital management feeling they had little control over them, for example:

*‘*These are adults they know what they should do’*.* (*Senior manager*).

*‘*Even if they are given a job description they will sign for them but later they will deny ever having received something like that…… job description cannot improve a person’s behaviour*’.* (*Senior manager*).

Though there were several key stakeholders involved in service delivery, there was limited involvement of the different parties in major decision-making processes. Absence of regular forums where working relationships could be discussed resulted in failure of the stakeholders to identify themselves with the aims of the hospital. A particular example was the poor institutional collaboration between KNH and UoN, also attributed by some to dissolution of the joint ‘Division of Paediatrics’ in 2004 that reportedly had the role of fostering good relationships:

*‘*Our relationship with UoN used to be good those days when we had Division of Paediatrics. It was a unifying body between university and KNH; we could discuss our working relationships… Division used to channel issues through MAC (*Medical Advisory Committee)…* But one of the hospital directors did not like MAC, it was a very powerful body that made changes happen…nowadays; we work like we have different interests’*.* (*Senior administrator, KNH*).

*‘….*KNH does not value our contribution and they don’t respect us, it is a system which is not working, they don’t invite us for meetings‘. (*a senior academic*).

*‘*Collaboration? For what? Do we need them (academics*)?’*- *KNH senior manager comments on the need to strengthen relationship between UoN and KNH.*

‘..one of the problems we have in KNH is poor communication, we can have team work only if there is good communication’ (*Nurse manager; audit feedback meeting*).

Poor communication limited knowledge sharing. One example was the limited use of research, much of it operational in nature, which was conducted within KNH by trainee paediatricians under the auspices of UoN. In fact, the majority of these projects were not even shared with the KNH management. Another example was inadequate communication of hospital policies and standard operating procedures (SOPs) that could have aligned health workers’ behaviour to the expected norms and common goals, which compromised teamwork:

Where are those SOPs? I have been in this hospital for many years (over 25 years) but I have not seen any SOPs. (*Senior academic; clinical audit feedback*).

#### Limited objective mechanisms for monitoring and evaluating quality of clinical care

Medical professionalism within KNH could be compared with the functional model [26]. Such a model provides doctors with considerable autonomy. It relies on self-regulation guided by commonly held (but not formally articulated) professional values that assume professionals will serve the best interests of the patient and adopt norms espousing this service orientation. Absence of more objectively assessed measures of good patient care meant inadequacies in self-regulation could arise and persist without notice. It was described by several paediatricians, in the following ways:

*‘*Supportive care in our ward is very poor. Nurses indicate they have given treatment, but if you ask the mum, you find that it is not true (*PO asked why this issue was not raised in the audit feedback meeting*..).. consultants know about nurses cheating that something has been done and they chart falsely on the treatment chart. So why do we have to say this while they (consultants) are quiet. They know the problems in that ward’. (*Trainee paediatrician*).

‘….when is your next audit? …A niece of a friend of mine was admitted in the wards with diarrhoea and vomiting and died on the third day. I felt sorry. We never think of the poor care we give our patients until one is affected directly. You see nowadays nobody cares’ (*Paediatrician*).

There were many examples suggesting that self-regulation was failing. For example, there was little effort to ensure adequate medical record keeping. Though medical notes are legal documents, we observed that they were not always labelled with a patients’ name, date and time, or signed by a clinician. The follow-up notes were often sketchy, not always in chronological order and results for investigations were rarely clearly documented. Some treatment charts were illegible with unauthenticated alterations. Despite revelation of these behaviours during audit feedback, these practices largely remained unchanged throughout the 18 months of the PAR although arguably, changes were within the power of the professionals.

#### Competing priorities

All the 25 paediatricians from KNH and UoN were salaried. However, within KNH there is a doctors’ plaza that is intended to encourage hospital specialists to have their private practice within reach of KNH while being an income generating activity for the institution, clearly sending mixed messages. Performance of paediatricians was further affected by the fact that there were apparently no explicit mechanisms for monitoring and evaluating either the quantity or quality of the services they offered. Interestingly what mattered seemed to be simply whether or not they ‘showed up’ during twice-weekly major wards rounds, as described below:

‘First we ensure attendance (*of ward rounds*) then quality. But people are busy elsewhere, no time for KNH. ‘(*Senior manager*).

‘Consultants do not do ward rounds (*problem marked in one of the wards*). They make technical appearances. Sometimes just report at 11 am and then go away. We have reported this problem and no action…on the day they are on call they are reminded to come for the ward round but they don’t come. (*Nurse Manager*).

#### Limited capacity for strategic planning

Hospital assessments during ETAT+ trainings revealed that the paediatric wards were ill-prepared to handle emergencies. Despite resuscitation of collapsed children being common, key drugs and equipment for resuscitation were often missing or kept in inappropriate places. For example in three wards, resuscitation couches were kept in the ‘procedure room’ while patients were resuscitated on ordinary beds. Although bag-valve-mask devices were available, their sizes were inappropriate for the age group 2-59 months, suggesting that the staff and managers were not aware of the specifications for equipment for paediatric resuscitation.

Despite congestion of patients on the wards, care of the seriously sick patients was not duly prioritized. Discharged patients (retained on the ward for non-payment of hospital user fees) comprising sometimes up to a third of ward patients, continued to receive injectable drugs and being reviewed regularly by clinicians and nurses. This was attributed to fear of patients dying while waiting to go home. Yet poor care revealed in the audits was largely attributed by staff to overcrowding of patients and staff being overworked as these statements suggest:

*‘*People (*nurses*) are not changing behaviour because they are overworked (*Ward ETAT+ coordinator*).

‘You see there are many problems, issue of overcrowding (*of patients on the ward*), how do we address it? Nurses are rebelling because of overcrowding’ (*Senior manager*).

‘The large number of discharges (*discharged patients retained on the ward due to non-payment of the user fee*) has brought the morale of staff down, people can’t work like this!’ (*Senior academic after a ward round*).

It was also found that ‘overworked’ staff often diverted their limited time from essential clinical work to performing tasks that could be automated or performed by less skilled personnel. Admission, discharge and billing services relied on manual, paper-based processes and the need to physically deliver documents from one place to another. These tasks were often done by the nurses. Similarly, there was delay in communication between the laboratory, radiology and pharmacy departments resulting in doctors having to physically ‘chase’ the results or other information from these service delivery points.

#### Inadequate management skills to introduce and manage change

From our observation, unwillingness to do things differently reflected a general negativism towards innovation and limited ability of the managers to articulate, supervise and guide change efforts, for example:

‘There is something wrong in this hospital. You want to improve care, so you introduce a change, people seem excited initially but then the steam dies off slowly. You see the hospital does not care, there is no supervision and so nobody cares’. (*Hospital staff*).

‘You know these people (*top-level managers*) say they are supporting us. But imagine they have not come to see what we are doing. They keep on saying that they will come. They only want to know what we are doing with the discharges (*discharged patients retained on the ward for non-payment*). It is frustrating’. (*Nurse commenting on the management’s supervisory support on the reorganization of ward lay-out*).

#### Hierarchical relationships

The relationship of the consultants with other staff in the hospital appeared to be a barrier to organizational learning. Passage of knowledge was largely unidirectional with the lower cadres being the recipients, rather than working as a team and drawing knowledge from the group. In some cases, paediatricians gave inappropriate information that was not questioned by the junior staff. This avoided conflict, though at the expense of patients’ safety:

‘I don’t want to hear those (*WHO steps for management of severe malnutrition*) steps. I want you to manage this child (*with diagnosis of marasmus*) as having failure to thrive so that we can give a holistic approach in the management’ (*Senior academic; ward round*).

Other times trainee paediatricians pretended to follow instructions from paediatricians but then gave the treatment they felt was correct, for example:

‘..don’t worry doc, I have done the right thing…. You know our consultant wants me to alternate 5% dextrose with Ringers’ lactate. So that is what I wrote on the treatment sheet during the round. But I am giving Ringers’ fortified with dextrose and potassium chloride. You see I have to appear to do what I am told’. (*Trainee paediatrician)*.

#### Paternalistic relationships

There was little effort made by the professionals to share information with patients or increase their understanding of their illness situation while in the hospital. Some caretakers neither knew the diagnosis nor the nature of treatment prescribed for their child. Doctors as well other health workers thus maintained their primacy in care of patients and protected their profession. This power imbalance made patients vulnerable because they were not empowered to engage constructively in their care; for example to question why their children did not receive treatment regularly, as illustrated here:

‘I told the nurse who was on night shift several times (*that the child missed treatment*), but she was just sitting (*doing paper work*) at the (*nurse’s*) desk. For me doctor, I just want my child to get well and I go home’. (*Parent, ward round*).

‘…problem is even attitude of the nurses. If the mother reminds the nurse to give treatment they would be ignored’. (*Nurse Manager*).

‘Actually, I agree yesterday most children did not get chloramphenicol and crystapen because most *(IV)* lines were tissued. So some nurses gave IM (*intramuscular*) crystapen but not to all children but they feared to give chloramphenicol… No, the doctor was not informed’. *(Nurse Manager; - ward round)*.

#### Inadequate adaptation of ETAT+ to the local context

Among all cadres there was deficiency of knowledge in some very basic procedures that were not the focus of ETAT+ (Table 5). In addition, KNH service delivery and monitoring tools such as vital sign observation charts, nutritional assessment forms and diet charts were outdated and did not permit staff to follow ETAT+ guidance. For example nutritional assessment forms did not include measurement of length/height and there was no mention of F75/F100 in the diet ordering forms.

**Table 5:** Processes of care and knowledge or skills incorrectly assumed to be sufficiently present among the KNH staff

**Process**	**Knowledge or skill observed to be deficient among ETAT+ participants**
Assessment of the key signs	Effects of illness on the physiology of the sick child that brings about the key signs.
	Perception of the health workers of the signs ‘inability to drink’ and intermediate levels of consciousness between a state of alertness and unarousable coma.
Assessing nutritional status	Measuring patients’ length/height
	‘(…*can we see your height measuring board?*). What is that? ….We don’t have one. (..*and what’s that?- pointing a height measuring board*). I don’t know, I have always seen it there’. *(Nurse giving responses in a rapid hospital assessment exercise*).
Treatment	Importance of administering drugs as prescribed and documentation of the same
Fluid therapy for dehydrated children	Incorrect but commonly used IV fluid for Plan C; Hartman’s Solution in 5% dextrose
	‘… yes we use Hartman’s in 5% dextrose for severe dehydration. We were told the blood sugar becomes diluted even if its e.g. 13 mmol/l after giving plain Hartman’s it drops quite low’. *(Junior clinician justifying use of 5% dextrose Hartman’s for Plan C during a CME).*
	Monitoring rate of administration and charting fluid chart.
	‘Gosh we did not know…….you mean we have been doing rubbish work. God forbid’. *(Nurse- during a CME on how monitor and chart intravenous fluid administration)*.
Monitoring of the sick child	Using serial respiratory and pulse rates to monitor patient progress and making clinical decision.
	‘If a nurse does not monitor patients’ vital signs what is she actually doing? *(Nurse A)* …Before I went for paediatric nursing, I could not interpret vital signs. I believe they are not monitored because people don’t see their value. *(Nurse B*)’.
Feeds for the malnourished and also NG feeds	Storage of feeds, approximation of daily feed requirement.

#### WHO pneumonia classification provided mixed messages

The ETAT+ classification of illnesses was based on WHO guidelines in use during the study period [27]. Thus, pneumonia was classified in order of severity as very severe pneumonia, severe pneumonia and pneumonia as opposed to the older WHO classification of severe pneumonia, moderate pneumonia and mild pneumonia respectively [28]. Both categories of severe pneumonia syndromes were however often perceived simply as ‘serious pneumonia’ and, contrary to the CPGs, considered as a single grouping worthy of treatment as ‘very severe pneumonia’.

## Discussion

The approach used in this research was participatory and sought to engage service providers as partners in the research process while aiming to explore ‘how things work’ in the KNH. We utilized naturalistic inquiry and participant observation made in real time. An interpretive and reflexive approach employed to analysis, which did not restrict us to a single level of analysis (individual or team or organization), helped us to engage with the complexity at the system level. We chose not to conduct formal, scheduled interviews to specifically explore (reported) attitudes and perceptions. Such interviews may have complemented the work reported although we feel the 18 months of detailed engagement and inquiry did provide us with an in-depth understanding of the perspectives of multiple stakeholders.

This PAR illustrates that it is possible to observe the action of individual health professionals at the time they are giving care to distressed patients, contrary to earlier reports [29]. These encounters are important because they are the final pathways through which CPGs ultimately affect the lives of the sick person. However, to understand the contents of such interactions the researcher needs expertise in the phenomena under study while experience in carrying out qualitative research is required to understand the social and interpersonal relationships observed. To the best of our knowledge, this is the first time an extensive ethnographical study (18 months), involving study of health worker-patient clinical encounters, has been reported from LICs.

The social science approaches helped articulate the range of professional behaviour in KNH, by revealing the rules governing both the individual and organizational behaviours. The long-term (18 months) ethnographic approach allowed us to go beyond what people say they do to see what they actually do (observed behaviour) while being alert to serendipitous discoveries. In addition, most of the study investigators had long lasting prior interaction with the study context. Our long-term immersion in the field provided a broader contextualisation of the situations the staff faced, and we feel we had a greater opportunity to elicit their perspectives and experiences of the ETAT+ implementation. It was also possible to relate events to antecedent conditions and to recognize the role prior experience can play, which was also informed by exploration of archival data on the history of KNH and University of Nairobi Medical School as linked institutions.

The research team comprised a mix of legitimate members of KNH and UoN, collaborators in the development of the CPGs and the ETAT+ course, persons in authoritative positions, an anthropologist and, a PO and facilitator with a medical background. It is likely that our working, experiences and epistemological perspectives have influenced our interpretation of the events. It is also likely that the composition of this team influenced the participants and the management’s actions. Nevertheless we were clearly unable to deliver a comprehensively successful intervention and had to re-think many of the assumptions we held about the institutions and implementation of ETAT+. To facilitate this and help avoid a biased interpretation we deliberately utilised a highly reflexive, iterative and long-term approach when trying to make sense of events and observations.

A lot of literature available on implementation of guidelines is from high-income countries whose contextual factors are different from those of LICs. For example, in high-income settings concerns of professional conduct and competence expressed in the media and political arena has prompted debate about the accountability of clinicians and professionals’ autonomy and led to a search for mechanisms to hold institutions and professionals accountable [30,31]. In addition, there has been a shift towards engaging patients as partners in decision-making and have their preferences considered [31,32]. This study reveals a different context. Thus, within KNH the health professionals appeared to exhibit paternalistic characteristics. They believed themselves to be trusted and the management considered them to be self-organizing. There were neither robust professional nor managerial accountability processes. With this background in mind, we discuss the PAR and the factors that shaped its evolution.

### The use of action research

ETAT+ was used to achieve variable personal and institutional goals that did not necessarily result in actual improvement of patient’s care. The multiplicity of meanings and goals linked to ETAT+ training resonates with the subjective interpretation of science by agents in other studies [33]. These results suggest that quality initiatives related to building capacity such as educational programmes, audit and feedback, problem analysis and action planning should not be treated as end-products. Rather they are parts of a process, whose real meaning emerges when the whole process is completed. Reporting success of *parts* risks losing sight of the *whole*; whole in this study being actual provision of quality care throughout an admission. Knowledge, therefore, should not be treated as a tangible thing, rather as an object that cannot be separated from its use [34].

Efforts to implement agreed solutions did not follow the orderly sequence suggested by linear models of implementation of quality initiatives, rather processes were evolutionary and context dependent. For example, inter-professional learning to enhance teamwork was a challenge as educational needs varied. Further, though it was our desire to have the staff and management actively engage in the PAR, both preferred to be engaged mainly through attending meetings and planning for action. Active implementation was largely left to few interested individuals, particularly the trainee paediatricians and those professionals who chose a leadership role. This resulted in a disjointed and *ad hoc* implementation processes and failure to complete the Plan-Do-Study-Act cycle, a finding documented in other action research [33]. Knowledge translation was therefore not smooth and encountered several problems related to the practices and competency of the hospital management and individual (or groups of) health professionals.

### Factors that influenced uptake of best-practices

A vision that is merely rhetoric fails to provide a sense of identity and to lay a foundation for organizational norms and structures [35]. In the KNH context, the vision failed to resonate with the reality and was unable to give direction to the managers and staff. In addition, the workforce and service organization were not commensurate with local morbidity and mortality patterns undermining organizational responsiveness to immediate societal needs.

This study also highlights the potential importance of professionalism. On one hand there is an outer observable behaviour of professionals that can be assessed by non-medical observers; this, we argue is what can be acquired through formal and codified knowledge. On the other hand is what we regard as *inner professionalism,* acquired through non-formal, un-codified and tacit knowledge [36]. Aspects of *inner professionalism* are probably opaque to non-medical researchers. This study teased out some nuances of *inner professionalism* such as the skill-practice mismatch and the inability of junior staff to challenge the practice of their seniors or share new knowledge inhibiting development of a learning organizational culture [37].

Many studies on implementation of best-practices focus on educational models for changing individual health professional behaviour (including our study). Our results revealed major organizational problems not directly addressed by our interventions. Rather the expectation is that organisations will be responsive to signals, such as audit feedback, that indicate the need for change. Achieving change may however require the building capacity to introduce change in an organization. For example, enabling the management to develop evidence-based strategic plans and policies, promoting objective mechanisms for monitoring service delivery, and fostering effective and timely communication that will help define and deliver desired standards of care. To achieve this, and arguably a key issue in our context, working to build effective relationships between groups and individuals may be more important than improving individual technical competence in such complex settings as KNH [38].

Poor hospital care in LICs has sometimes been attributed to lack of knowledge and resources [1,39,40]. While this may be true for complex or chronic diseases, we suggest in this large referral hospital, poor care for common acute childhood illnesses is often also due to poor planning, limited critical evaluation of service provision, and poor self-regulation among the professionals who are currently the *de facto* service leaders in the absence of engaged management. In fact to implement the CPGs, that are the focus of this study, required relatively few basic resources (with the exception of adequate nursing staffing). Solutions to poor care may therefore need to be more nuanced than simply calling for additional resources and may need to address fundamental institutional, organisational and professional factors as part of a broader change management process occurring within a complex environment.

## Conclusions

Work of the type we have undertaken is rarely reported from LICs but echoes findings from higher income settings [41,42]. This work strongly suggests that educational interventions, often regarded as quick-fixes to improve care in LICs, may be necessary but are unlikely to be sufficient to truly deliver improved services. We found the PAR approach a valuable mechanism for exploring our fieldwork context, adapting and implementing evidence-based care. It also provided a basis for developing an understanding of the breadth, duration and effort that are likely to be required to change service delivery in a major health institution. Changing such institutions is however of considerable importance. Major teaching hospitals may contribute disproportionately to the culture of health care practice in countries such as Kenya where three quarters of all Kenyan medical graduates train in our study hospital. Failure to imbue young professionals with appropriate practice skills and professional values may result in long-lasting health system problems.

## Abbreviations

CMEs: Continuous medical education sessions; CPGs: Clinical practice guidelines; ETAT+: Emergency, triage, assessment, treatment PLUS admission care; KNH: Kenyatta national hospital; LICs: Low-income countries; PAR: Participatory action research; PO: Participant observer; QIs: Quality indicators; UoN: University of Nairobi.

## Competing interests

The authors declare that they have no competing interests.

## Authors’ contributions

GI conceived the idea for this study and its design with advice from ME. ME obtained the funding for this project. The study hospital and ME provided financial support for the guideline dissemination. GI, HK, CM, DM DG, SM and ME guided/facilitated the quality initiatives in the action research. GI was responsible for data collection. GI, ME, DZ and AG were responsible for data analyses and interpretation. GI prepared the initial draft manuscript. All authors reviewed the draft manuscript and provided input to and approval for the final version of the report. All authors read and approved the final manuscript.

## Authors’ information

CM, HK, DM, ME and GI are paediatricians and legitimate members of KNH and UoN. ME is a Senior Research Fellow with Wellcome Trust, he conceived and was instrumental in development of the CPGs and ETAT+. GI participated in the development of the guidelines and ETAT+ training. CM and DM were head of KNH and UoN departments of paediatrics respectively. AG is an anthropologist. DZ is an epidemiologist. SM is a paediatrician and a senior officer in the Ministry of Health.

## Pre-publication history

The pre-publication history for this paper can be accessed here:

http://www.biomedcentral.com/1472-6963/14/59/prepub
